# Surgical stabilisation versus external coaptation for treatment of metacarpal/metatarsal bone fractures in dogs

**DOI:** 10.18849/ve.v10i3.712

**Published:** 2025-07-21

**Authors:** James Phillip+

**Keywords:** CANINE, CONSERVATIVE MANAGEMENT, DOGS, METABONE FRACTURE, METACARPAL FRACTURE, METATARSAL FRACTURE, SURGICAL MANAGEMENT, TREATMENT OUTCOMES

## Abstract

**PICO question:**

In dogs with metacarpal and metatarsal fractures, does open reduction and surgical stabilisation compared to closed reduction and external coaptation (non-surgical stabilisation) lead to an improved likelihood of bone union and resolution of lameness?

**Clinical bottom line:**

**Category of research:**

Treatment.

**Number and type of study designs reviewed:**

Five retrospective studies that directly compared surgical intervention to conservative management of metabone fractures were critically reviewed.

**Strength of evidence:**

Weak.

**Outcomes reported:**

External coaptation may lead to successful clinical outcomes when there is minimal displacement of the metabone fractures but malunion may be more likely when using external coaptation. Clinical outcomes may be similar in many of these patients whether we treat surgically or non-surgically, but surgery seems to be more prudent when there is a high degree of displacement. It would seem that cases that require surgery are more likely to have a higher complication rate, but this is likely due to their more complicated nature. Potentially bone plates are superior to intramedullary pinning if surgery is chosen and open reduction and surgical intervention may be more likely to result in radiographic synostosis however this may be clinically insignificant regarding functional outcome.

**Conclusion:**

Strong evidence-based treatment guidelines are lacking in this area so surgical intervention of metabone fractures cannot be definitively recommended over closed reduction and external coaptation in the current literature review.

## How to apply this evidence in practice

The application of evidence into practice should take into account multiple factors, not limited to: individual clinical expertise, patient’s circumstances and owners’ values, country, location or clinic where you work, the individual case in front of you, the availability of therapies and resources.

Knowledge Summaries are a resource to help reinforce or inform decision-making. They do not override the responsibility or judgement of the practitioner to do what is best for the animal in their care.

## Clinical scenario

A four year old male Cocker Spaniel (14 kg) is involved in a road traffic accident (RTA). He is stable but investigations find that he has mid diaphyseal fractures of all four of his left metacarpal bones with moderate displacement and marked instability. The client expresses some financial concerns and is keen to avoid surgery but wants to do what is best for the dog. The veterinarian must decide whether the case requires surgical stabilisation or whether it could be managed conservatively with closed reduction and external coaptation.

## The evidence

The strength of the evidence that directly compares conservative to surgical management of metabone fractures is weak. Five papers met the inclusion criteria (Bellenger et al., 1981; Kapatkin et al., 2000; Kornmayer et al., 2014; Manley, 1981; Muir & Norris, 1997). Three papers were published in the twentieth century (Bellenger et al., 1981; Manley, 1981; Muir & Norris, 1997) while the most recent papers also featured cases treated in the twentieth century (Kapatkin et al., 2000; Kornmayer et al., 2014). All five papers are retrospective studies with relatively small case numbers (< 50 cases) and unconvincing follow up data, lacking full veterinary assessment of outcome, with the expection of Kornmayer et al. (2014) which looked at 100 cases with full radiographic and clinical follow up. None of the studies involved randomised treatment selection and surgical intervention techniques greatly varied. Two of the earlier studies (Manely, 1981; Muir & Norris, 1997), which recommended surgical intervention over conservative management in most cases, had relatively poor outcomes regardless of treatment chosen,compared to the two more recent papers (Kapatkin et al., 2000; Kornmayer et al., 2014) which overall had very good outcomes with both treatment modalities, so there is a significant degree of inconsistency with relation to prognosis in general. The two more recent papers perhaps contained the strongest evidence with Kornmayer et al. (2014) looking at 100 cases with metabone fractures that had complete radiographic and clinical follow up, and Kapatkin et al. (2000) which looked at 25 dogs, all of which met the empirical criteria that is widely accepted to warrant surgical intervention. Of these 25 cases 16 were managed conservatively and 9 were managed surgically.

## Summary of the evidence

**Bellenger et al. (1981):** Fixation of Metacarpal and Metatarsal Fractures in Greyhounds **Aim: **To retrospectively assess the aetiology and distribution of metacarpal and metatarsal fractures in racing greyhounds and their ability to return to training and racing following treatment with either external immobilisation or internal fixation.

Population:	Mix of male and female dogs presented between 1974–1980 University of Sydney Hospital (Australia) with metacarpal and metatarsal fractures aged between 3–96 months.
Sample size:	33 dogs. 23 greyhounds. 10 dogs of other various breeds – these were excluded from the study by the original author following assessment of initial presentation/mode of injury (mainly external trauma) and fracture location with number of metabones fractured so are not analysed further in this Knowledge Summary.
Intervention details:	1 case was euthanised. 8 cases with single metabone fractures were treated conservatively with Plaster of Paris casting (POP) for 14–21 days. Internal fixation was used in 14 dogs. Screws alone used in 8 dogs in lag screw method. 2 cases had screws and K wires. One case with multiple fractures was repaired with combination of screws and intramedullary (IM) pinning. 3 cases were repaired with screws and plates due to comminution. All surgical cases had POP casts applied 48–72 hours post surgery which were removed after 2–3 weeks. All dogs were evaluated for implant removal 8–12 weeks postsurgery. 12 weeks rest was recommended for 12 weeks post implant removal.
Study design:	Retrospective case series.
Outcome Studied:	Mode of injury: 22/23 greyhounds were track injuries. Remaining case not specified. Fracture location and configuration as per radiographic findings: 22 cases involved metacarpals vs one case involving a metatarsal fracture. 21 cases were single bone fractures and 2 were multiple. 14 were diaphyseal and comminuted. 7 cases were oblique. 2 were transverse. Final radiographic follow up was performed in 16 cases ranging from 0–126 weeks postoperatively. 14/16 were surgically managed. 2/16 were conservatively managed (POP). Time frame since surgery of implant removal was recorded ranging from 6–20 weeks following surgery. Ability to return to racing and racing performance were the main focus of outcomes studied.
Main Findings (relevant to PICO question):	Radiographic follow up for 16 available cases: The 14 surgically managed fractures were recorded as either, well reduced (n = 2) healed (n = 2), healed with callus (n = 2), healed with minimal callus (n = 3) or healed without callus (n = 5). The 2 conservatively managed cases (POP) with radiographic follow up were recorded as callus (n = 2). Clinical follow up with respect to ability to return to racing. 1 case was still being rested at follow up (surgical case). 2 cases were lost to follow up (both conservatively treated with POP). 3 cases were retired without trialing (1 surgical case, 2 conservatively managed cases (POP) ). 6 cases were retired after trialing, but it is suggested these were slow young dogs – no indication retirement was related to the injury (4 surgical cases and 2 conservatively managed (POP)). 2 cases were trialed and developed further injury (both surgical cases). 4 cases had less than 10 race starts following injury (3 surgical cases and 1 conservatively managed case (POP)). 4 cases raced with more than 10 starts each (3 surgical cases and 1 conservatively managed case (POP)). Out of the remaining cases that were either lost to follow up, retired or developed further injury after trialing – 8 were surgical and 5 were conservatively managed. Radiographic follow up revealed healing in 12/14 of the surgical treated cases and the two that were not reported to have healed were radiographed too early in the healing process but showed the fracture to be reduced. Radiographic follow up of the 2 conservatively managed cases revealed callus formation but did not state whether they had healed.
Limitations:	Retrospective case series. The study was published over 50 years ago with cases taken from 7 years prior to this. Small case numbers. No complications recorded other than in the 2 cases of further injury. Treatment selection was not stated to be randomised. All surgically stabilised cases received post operative external coaptation in the form of POP so it would be difficult to compare the results against POP alone. No measure of residual lameness or clinical outcome at follow up. Only radiographic follow-up is available for 2 of the conservatively managed cases. Surgical techniques implemented in these cases may be outdated with more modern types of osteosynthesis not included in the study. 21/23 (91%) of the fracture cases involved only single metabone fractures. 20/23 (87%) of the cases involved only fractures of either the second or fifth metabone. These injuries are very specific track injuries and therefore comparison or use of the information may not be applicable to the general pet population.

**Kapatkin et al. (2017):** Modified tube gastropexy using a mushroom-tipped silicone catheter for management of gastric dilatation-volvulus in dogs **Aim: **To retrospectively compare the outcomes of metacarpal and metatarsal fractures in dogs treated either surgically or conservatively.

Population:	Dogs weighing between 1.4–35 kg with fractures in the metacarpals or metatarsals in one leg (1986–1996) all meeting the metabone empirical “surgical criteria” of one or more of the following: More than 2 fractured metabones in the same leg. Fractures of the 2 weight bearing bones in the same leg. Articular fractures. All cases came from a single institution, the University of Pennsylvania (USA).
Sample size:	25 dogs.
Intervention details:	Fracture displacement ranged from none to severe and there were two cases of open fracture. No dogs had other orthopaedic trauma or disease. All owners were encouraged to have surgical fixation due to aforementioned recommendations. Follow up was 9–68 months. Surgical or conservative treatment was assigned based on each owner’s decision, which divided the cases into 2 groups. Surgically treated (n = 9): 5 intramedullary (IM) pins 1 external skeletal fixation (ESF) 1 screws 1 bone plating 1 with figure of 8 wiring. All surgical treated cases were supplemented with a modified Robert Jones Bandage (RJB) and orthoplast splint for 4–6 weeks. Conservatively treated (n = 16): Splinted dressings (modified RJB and orthoplast splint). Sometimes an aluminium walking bar.
Study design:	Retrospective case series.
Outcome Studied:	Outcomes were determined with a combination of preprepared telephone questionnaires (n = 25) and recheck examinations within the hospital (n = 19) to determine the dog’s clinical outcome at follow up times of 9–68 months after injury. Scores were given as: 1 (Completely normal) 2 (Imperfect result).
Main Findings (relevant to PICO question):	The outcome was not statistically affected by surgery or conservative treatment. Owners/clinicians rated perfect results in 9/16 (56%) of conservatively managed cases and 7/9 (77%) of surgically managed cases. To keep scoring consistent if either client or vet gave the patient an imperfect score, they were allocated an overall score of a 2 (imperfect result). All 9 surgical cases had clinician rechecks whereas only 10/16 conservatively managed cases had clinician rechecks. Age and weight were not deemed statistically significant in either group though the conservatively managed group was on average younger and lighter (28 months vs. 42 months and 12 kg vs. 16 kg). Fracture displacement was not statistically significant between both groups. 2 dogs in each group had at least one articular fracture and all four cases recovered completely so this was not deemed statistically significant. Fracture configuration (complete vs. comminuted/open vs. closed) had no significant influence on outcomes in either of the two groups, however there were only two open fractures. The number of metabones fractured also had no significant statistical influence on outcomes in either group. The conservative group consisted of cases with 2–4 metabone fractures and the surgical group consisted of cases with 2–4 metabone fractures. Overall recovery time was longer in the surgically treated group: conservative – mean 7.1 weeks, median 7 weeks; surgical – mean 27.6 weeks, median 12 weeks. Surgical implants required removal in 4/5 IM pin cases and the ESF case. Reasons not reported.
Limitations:	Retrospective case series are a weaker form of study regarding strength and quality of evidence. Surgical vs. conservative treatment was assigned based on the owners’ decisions so it was not a randomised or blinded study. The predicted success of the fracture management choice still could have been influenced by the surgeon even though all cases were recommended to have surgery. The cases included in this study are from between 28–36 years ago. Advances in surgical treatments have progressed in this time. Small sample size. The significance of open vs. closed fractures could not be assessed due to only one open fracture in each group. Compliance with conservative group was reported to be low with regard to splint changes and hospital follow up in person which could have affected outcomes. Studies relying on owners making the final assessments can be highly inaccurate. Some of the follow up in person veterinary assessments in the conservative group were reported, by the author, to be too early in the recovery period to give an accurate final outcome in these cases. The surgical intervention technique was not standardised so comparing surgery to conservative management is difficult given there can be very varied outcomes between different surgical approaches. Follow up radiographic assessment was not consistently performed.

**Kornmayer et al. (2014):** Long-term prognosis of metacarpal and metatarsal fractures in dogs. A retrospective analysis of medical histories in 100 re-evaluated patients **Aim: **To retrospectively evaluate cases of metacarpal and metatarsal fracture in dogs with complete clinical and radiographic follow up to determine the long term prognosis.

Population:	Dogs of mixed breed and age with complete clinical and radiographic follow up following metabone fractures within 4months–14 years post injury. Cases from 1990–2007 from a single centre. 37% of animals were under 1 year old and 41% were under 2 years old. Mean age was 2.6 years.
Sample size:	100 dogs.
Intervention details:	55/100 (55%) of the fractures were < 50% displaced. 14/100 (14%) of the fractures were 50–100% displaced displaced grade 2. 31/100 (31%) of the fractures were > 100% displaced. 85/100 (85%) of fractures affected the body of the metabone. 84/100 (84%) of fractures were closed. Cases were divided into 3 groups: Conservative management. Surgical management. Combination of both surgical and conservative management. Treatment selection was made on a case by case basis. Group 1 (conservative management): 67 dogs (68 limbs) with mildly displaced, non-reconstructible multiple bone fractures, physis fractures, or multiple bone fractures which could undergo closed reduction under general anaesthetic and external coaptation. Some with open fractures underwent debridement, ± wound closure. A synthetic splint and bandage was applied for an average of 6 weeks (range 4–12 weeks). The bandages were changed weekly. Group 2 (surgical treatment): 25 dogs (25 limbs) with severely displaced fractures of single bones, re-constructible articular fractures, and multiple bone fractures particularly those involving third and fourth metabone. Dowel pinning and standard dorsal bone plating were most commonly used but there were cases of external fixation, lag screws and medial/lateral bone plates. All cases had a Robert Jones Bandage (RJB) applied for 4–8 weeks, changed weekly. External fixators and most bone plates were removed when healing was radiographically diagnosed. Group 3 (combined surgical and conservative treatment): 8 dogs with multiple fractures of which not all could be surgically stabilised due to comminution, short bone fragments, or due to specific skin wounds. These cases had a combination of surgery and external coaptation similar to patients in group 1 i.e., synthetic splinted support dressings changed weekly.
Study design:	Retrospective case series.
Outcome Studied:	Outcomes evaluated at average time frame of 4 years post initial presentation. Final radiographic outcomes were interpreted by two investigators using orthogonal views of the affected and contralateral limb. Clinical/functional outcomes were graded as present or absent. 16 dogs were graded by more than one clinician, while 84 dogs were graded by a single clinician. 15 cases were analysed using computed gait analysis using treadmill with force plates owning to patient/client compliance.
Main Findings (relevant to PICO question):	The main findings highlighted below are those relevant to the PICO question. Fracture classification, degree of displacement, metabone number, location on bone and which limb with relation to complications were observed within the study but not reported in detail here. Radiographic outcome reported in respect to complications noted on assessment: Group 1 (conservative treatment): 2/67 (3%) dogs showed early complications of delayed union radiographically. At final radiographic follow up; 9/67 dogs had malunion (13%). Synostosis occurred in 5/67 dogs (7%). Osteoarthritis (OA) was seen in 2/67 dogs (3%). Non union was present in one bone of 1 dog (that had third to fifth metatarsal bone fractures). Group 2 (surgical treatment): 3/25 (12%) dogs developed early complications. 2 developed osteomyelitis and implant loosening. One of which had open shaft fractures ofmetabones (second to fifth) treated with dowel pinning. The other had base fractures of metabones (second to fifth) repaired with cross pins. The third dog had open metabone (second to fifth)fractures and was treated with an external skeletal fixator. Delayed union occurred. At final radiographic follow up; 3/25 (12%) dogs had malunion (12%). 3/25 (12%) dogs had synostosis. Group 3 (combination of conservative and surgical treatment): 3/8 (38%) exhibited complications. 2 cases had implant loosening without bone healing impairment. Both had second metacarpal treated with bone plate and the third metacarpal was treated with external coaptation. The third case had delayed union. This case had open fractures of second to fifth and was repaired with oversized plates. To prevent non union revision surgery to reduce implant size and apply cancellous bone autograft was required. At final radiographic follow up; 2 dogs had malunion (25%). 1 dog showed evidence of OA. Synostosis occurred in 3 dogs. Synostosis in all cases seemed more frequent in proximal metabone fractures and multipart fractures. Functional outcome: Clinical assessment of resolution of lameness: Group 1 (conservative treatment): 65/67 (97%) dogs were free of lameness. Lameness was due to malunion in one case and severe soft tissue injury in another leading to OA. 10 cases had bandage associated problems during recovery – sores, dermatitis, etc. which required treatment but had no significant bearing on final outcomes. Group 2 (surgical treatment): 24/25 (96%) dogs free of lameness. Lameness was due to implant loosening requiring early implant removal and malunion of third metabone. Group 3 (combination of conservative and surgical treatment: 8/8 (100%) dogs free of lameness. Computed gait analysis matched up with clinical assessment for most cases (12/15) but found subtle lameness not detected on visual examination in 3 cases. Statistical results: Final outcomes were generally very good in this paper with only 3/100 (3%) of dogs having lameness reported, 1/100 (1%) having a non union and 3/100 (3%) developing radiographic signs of osteoarthritis. Overall there was a 14/100 (14%) rate of malunion and 19/100 (19%) rate of synostosis but these two complications had no bearing on functional outcome. Synostosis was statistically more common when surgical intervention was involved in terms of fractures of three to four metabones compared to conservative management. Despite a statistically higher incidence of synostosis in surgically treated patients there was no significant difference in outcome between conservative vs. surgically managed cases. The incidence of malunion, non union or OA and functional outcomes were not statistically different in the groups. There was no significant difference in outcomes between metacarpal or metatarsal fractures within the 3 groups; however, the study showed that if complications occurred, this would result in an increased rate of synostosis and malunion. This seemed truer in the metatarsal bones and malunion of the fifth metatarsal bone was more prominent. Complications were also more likely to occur when dealing with open fractures, oblique and comminuted fractures of the base, severely displaced fractures, and when surgery was required. This again was seen more frequently in the metatarsus. No correlation between potential influencing factors and lameness was detected, because lameness was actually rarely found at the final follow up. No statistical conclusion can be made to recommend surgical vs. conservative management but it would appear good outcomes can be achieved from either approach.
Limitations:	Retrospective case series are a weaker form of study regarding strength and quality of evidence. Case treatment was not randomised so it could be argued that groups 2 and 3 were made up of more challenging cases with more severe fracture displacement and therefore comparable outcomes to group one would support the use of surgical intervention in said cases. There are many variations of fracture type in this region with regard to number of metabones, location of the fracture and degree of displacement so it is difficult to interpret the data. There were variable re-evaluation periods in patients so we can draw no conclusions to whether surgery or conservative management in these cases leads to earlier or more prolonged return to function even though long-term outcomes appeared to be favourable. There were discrepancies between the subjective assessment of lameness by clinicians and the vertical ground reactions in 3/15 cases that underwent computed gait analysis so this may indicate a clinician bias or difficulty in seeing subtle persistent lameness’ at long-term follow up of these cases. This could mean that the percentage of successful outcome could be falsely high.

**Manley (1981):** Distal extremity fractures in small animals **Aim: **To assess the outcomes of distal extremity fractures in dogs treated either conservatively with external coaptation or with surgical stabilisation and to make suggestions based of these for future treatment selection.

Population:	Dogs admitted at a single institute between 1978–1980, with distal extremity fractures, in equal numbers of forelimb and hindlimb involved.
Sample size:	43 dogs.
Intervention details:	12 cases did not meet the inclusion criteria and so only the 31 cases of metabone fractures with follow up data will be reported on this table. n = 31 dogs (6 single bone fractures, 25 multiple bone fractures, 15 fractures involved articular components). Conservative management by external immobilisation (n = 20 dogs) included either Robert Jones bandage (RJB), Mason Meta splint, casting and Thomas splint. Surgical management (n = 11 dogs) included either singularly or with combinations of intramedullary pins, single cerclage wire, tension band plates, interfragmentary compression with screws, internal fixation with a plate. Surgical intervention was recommended when there was marked fragment displacement or involvement of 2 or more bones and if there was articular involvement.
Study design:	Retrospective case series.
Outcome Studied:	Nature of fracture, treatment assessment, and long-term outcomes. Outcomes were determined via a client questionnaire encouraging critical evaluation of their dogs 4–26 months post treatment. Any evidence of residual lameness or swelling associated with the fracture or its treatment was noted as a complication and an unsatisfactory outcome was assigned. No veterinary assessments of these cases to correlate client perception were reported but 2 cases did have revision surgeries. Complications of malunion (n = 3) and osteomyelitis (n = 3) were also reported thus veterinary assessment must have been present for these cases.
Main Findings (relevant to PICO question):	Overall, complications occurred in 10/11 surgically treated cases with 8/11 having persistent lameness. Complications occurred in 11/20 of the conservatively treated cases and 8/20 had persistent lameness according to the questionnaire follow up. It is not specified how many of the cases had a follow up in person veterinary assessment. 6 cases had one single metabone fracture: 5 were treated conservatively with 3 developing complications (residual lameness, osteomyelitis). 1 was treated surgically and this developed complications (arthrosis and persistent lameness). 25 cases had 2 or more metabone fractures: 15 were treated conservatively. 5/15 conservatively treated cases had persistent lameness. 9/15 developed complications. One refractured after splint removal, one developed malunion requiring corrective osteotomies, and one required amputation of a digit. 10 were treated surgically. 7/10 surgical cases had residual lameness. 9/10 developed complications including malunion, malalignment, persistent drainage, or osteomyelitis. The fracture configurations all varied, and the complications were not statistically affected by the location of the fracture. The degree of fragment displacement and involvement of articular surfaces was a predictor of complications post treatment. 15 cases involved articular surfaces. 13/15 developed complications (residual lameness, osteomyelitis). Out of the 2 that were treated without complication one was conservative and one was surgical. None of the fractures with minimal displacement had unsatisfactory outcomes whereas 25/29 (86%) of all fractures reported in the paper with marked displacement had unsatisfactory outcomes. Unsatisfactory outcomes were also reported in 13/15 (87%) of those with articular involvement.
Limitations:	Retrospective case series are a weaker form of study regarding strength and quality of evidence. The methods of assessing outcome were poor. Client questionnaires – although useful in large numbers (registries) – can be unreliable and biased. There was also no access to the questionnaires for evaluation. Not every case had veterinary assessment and follow up. Patient data that may have had a bearing on the outcomes of the cases was absent e.g. the age and weights of the patients were not recorded. Whether the fracture was open or closed was recorded but the concurrent soft tissue damage or nature of injury was not. Modern surgical repair methods are superior to the techniques implemented during the years of this study, so to draw conclusions about the outcomes of the surgical treated cases is difficult. The study is now over 40 years old. Sample size was relatively small. Surgical intervention versus conservative management was not randomly selected so there may have been a bias to only choose conservative management in more simple fractures.

**Muir & Norris (1997):** Comparison of the Recurrence Rate of Gastric Dilatation With or Without Volvulus in Dogs After Circumcostal Gastropexy Versus Gastrocolopexy **Aim: **To retrospectively compare the clinical and radiographic outcomes in dogs with metacarpal and metatarsal fractures to ascertain whether fracture reduction would be improved with open reduction and internal fixation versus external coaptation.

Population:	Dogs with metabone fractures aged 2 months–10 years with a body weight of 1.8–42 kg. All dogs were from a single institute (The University of California, Davis, Veterinary Medical Teaching Hospital) over a 9-year period.
Sample size:	37 dogs.
Intervention details:	8 cases were RTA and 6/8 had other additional fractures of pelvis or other long bones. There were no ipsilateral metabone fractures in any dog. 26 of the fractures were acute(presented to the institute within 10 days of injury) and 11 were chronic (presented between 2 weeks and 10 months post injury). 23 metacarpal fracture and 14 metatarsal fractures: 9/37 (24%) one metacarpal fracture. 6/37 (16%) 2 metacarpals fractures. 7/37 (19%) 3 metacarpal or metatarsal bone fractures. 15/37 (41%) had 4 metacarpal or metatarsal bones fractures. Conservatively managed with exercise restriction only – 2/37: 1 with 1 metacarpal bone fracture. 1 with 4 metacarpal bone fractures. Conservatively managed with external coaptation(type not specified) – 24/37 dogs: 6 with 1 metabone fracture. 5 with 2 metabone fractures. 4with 3 metabonefractures. 9 with 4 metabone fractures. Surgical management – 11/37 dogs: 1 dog had a proximal chip fracture of one metabone. 1 had 2 metabone fractures along with a luxation of the carpometacarpal joint. 3 with 3 metabone fractures. 6 with 4 metabone fractures. Surgical techniques: 1 case with 4 metabone fractures was stabilised using k-wire cross pins across two of the metabones combined with external coaptation. 1 had intra-articular cross pinning and external coaptation due to luxation of carpometacarpal joint and comminuted metabone fractures. Intramedullary (IM) pining with external coaptation was used in 2 dogs (one with 3 metatarsal fractures and one with 4 metacarpal fractures. 6 dogs (2 with 3 metabone and 4 with 4 metabones) had bone plates. 5/6 had supplementary external coaptation applied. 2 dogs had bone screws placed.
Study design:	Retrospective case series.
Outcome Studied:	Pre- and post-treatment radiographs (minimum of lateral and dorso-palmerviews) measuring displacement and alignment recorded as improved or not improved post treatment. Displaced vs. non-displaced were categorised as less than 75% vs. at least 75% bone end alignment. Treatment outcome – healing of fracture radiographically and development of complications.
Main Findings (relevant to PICO question):	2 cases were chronic fractures managed with activity restriction, both were lost to follow up. Outcomes of cases conservatively managed with external coaptation (n = 24): 10 of these cases were lost to follow up. Progressive healing occurred in 13 of these dogs. Delayed union occurred in 1 dog. Fracture alignment improved in 5 dogs and did not improve in 5 dogs. Outcomes of surgically managed cases (n = 11): One case treated with intra-articular cross pinning failed and PCA was required. Progressive healing occurred in both dogs treated with IM pinning but malalignment with an element of non union was present. Progressive healing occurred in all 6 dogs treated with bone plates. 3 had improved fracture alignment but the other 3(that were chronic fractures) did not improve. Both cases treated with bone screws alone, had persistent lameness and required screw removal – both went on to progressive healing. The only firm conclusion from the paper was that distal metabone fractures are more likely to be displaced and have axial malalignment. External coaptation for fractures of 1–4 metabones usually resulted in progressive healing, however, if malalignment was present prior to placement of external coaptation this malalignment only improved in 5/10 cases (50%). This method should therefore be limited to minimally displaced proximal fracturesas distal fractures are more likely to result in malalignment and external coaptation is not adequatefor correcting this. Bone union did not occur in 3 dogs. 1 case had 4 metabone fracture treated with IM pinning and 1 case that had multiple open fractures repaired with IM pins. The third case was a conservatively managed third metacarpal bone fracture. In this study, bone plating (n = 6) corrected malalignment in 3 acute fracture cases but not in the 3more chronic fracture cases. Progressive healing occurred in all cases but there was no comment on clinical lameness.
Limitations:	Retrospective case series are a weaker form of study regarding strength and quality of evidence. The study is over 25 years old now – surgical intervention techniques have improved in this time. Small sample size. 12/37 cases from the conservatively managed groups were lost to follow up. Treatment was not randomised. No clinical assessment of ongoing or long-term lameness – focus was more on radiographic improvement of malalignment and bone union. No set observation time for re-assessment reported. No time frame set for their determination of non-union. Modern surgical techniques (small locking plate systems) were not assessed in case treatment and may be superior to surgical methods in the paper.

## Appraisal, application and reflection

The approach to treating fractures of the metabones (metacarpals and metatarsals) is a controversial topic. Generally accepted guidelines in the veterinary field would suggest that conservative (closed reduction and external coaptation) is only appropriate in non or mildly displaced fractures of 1–2 metabones and only if at least one of the main weight bearing metabones (third and fourth) is intact. In fractures involving; the articular surfaces, more than 2 metabones, both the third and fourth metabones, severely displaced or comminuted fractures, open fractures, and those of large breed or athletic dogs, fracture management should involve open reduction and surgical intervention as described by Manley (1981) and in textbooks such as *Handbook of Small Animal Orthopaedics and Fracture Repair* by DeCamp et al. (2016) , and *Small Animal Surgery* by Fossum (2007). When we look at the literature it has been suggested that these guidelines are extrapolated from older human recommendations and empirical in nature in the veterinary field (Kapatkin et al., 2000). The PICO question above was posed to see if there was improved likelihood of bone healing and resolution of lameness when comparing outcomes of metabone fractures that are treated conservatively versus those treated surgically to help aid clinical decision making.

Many papers were found relating to possible treatments of metabone fractures but there were only 5 papers (Bellenger et al., 1981; Kapatkin et al., 2000; Kornmayer et al., 2014; Manley, 1981; Muir & Norris, 1997) found in this literature search that specifically addressed the PICO and directly compared conservative and surgical treatment of metabone fractures. None of these papers were randomised studies and often treatment selection was determined by clinician judgment or client preference, often influenced by financial circumstances. All studies were retrospective case studies. Two of these were not accessible in English, so their contents could not be evaluated.

Manley (1981) looked at 35 cases of metabone fractures but only 31 were available to follow up with an observation time of 4–26 months. Twenty-one cases were treated conservatively and 10 were treated surgically. The choice of treatment was not randomised and generally the treatment was based on the established guidelines highlighted above. In this study 8/20 (40%) of the conservatively treated group were lame at follow up and 8/11 (73%) of the surgically treated cases were lame at follow up. Complications occurred in 11/20 (55%) and 10/11 (91%) of cases respectively but were higher in fractures that were more severely displaced and those involving the articular surface. Some surgical interventions also involved subjectively inadequate repair techniques with cerclage as a sole stabilisation agent, intramedullary (IM) pins that were too small compared to the medullary canal and pin ends that were left protruding into joints. A statement in the paper claims that external coaptation in cases of marked displacement can have “disastrous results” but it only pointed to one particular case as an example of this and it also concluded that if external coaptation does not extend beyond the carpus or tarsus (like the one in this particular case) one cannot hope to achieve appropriate stabilisation. Interestingly the number of bones fractured did not seem to be negatively affected by the use of external coaptation so perhaps the rules regarding external coaptation and the number of metabones fractured is not actually that significant in cases with no major displacement. The outcomes in this paper are much less favourable than more recent studies (Kapatkin et al., 2000; Kornmayer et al., 2014) and the surgically managed cases were generally cases that had more complicated fractures so this study cannot truly assess whether open reduction and surgical stabilisation compared to closed reduction and external coaptation (non-surgical stabilisation) would lead to an improved likelihood of radiographic bone healing and resolution of lameness.

Muir & Norris (1997) followed the same train of thought as Manley and concurred with the assessment that external coaptation was best reserved for minimally displaced fractures of 1–2 metabones; however, the data to support this was lacking in the paper. Their paper looked retrospectively at 37 dogs with metabonefractures (23 metacarpal and 14 metatarsal). Two cases were chronic fractures managed with activity restriction. Both were lost to follow up. Twenty-four cases; six with 1 metabone fracture, five with 2 metabone fractures, four with 3metabone fractures and 9 with 4 metabone fractures had external coaptation. Ten of these cases were lost to follow up and 13/14 remaining cases were noted to have progressive healing; however, it was not stated which of the fracture configurations these 13 cases belonged to. The fourteenth case developed a delayed union. It was noted that the fracture alignment improved in five of these dogs; however, in five there was no improvement to alignment despite evidence of progressive radiographic healing. Out of the 11 dogs treated surgically, that were available to follow up, all had evidence of progressive radiographic bone healing at follow up but two were persistently lame due to loosening of screws which were then removed. This unfortunately was the only mention of clinical lameness assessment in all the cases studied. The only statistical conclusion drawn from the paper was that there was marked displacement (more than 75% of the metabone fractured bone ends) in 71% of fractures of the mid-distal regions of the metabones compared to 49% of the proximal bones. Therefore, mid-distal fractures were more likely to result in malunion without surgical realignment. In these cases of marked displacement and malalignment, particularly when involving 3-4 metabones, Muir and Norris (1997) suggested that the best way to treat these is with small bone plates, not with IM pinning or external coaptation and this was based on malalignment improvement in 3/6 cases that matched this criteria within the study. The author implied that the reason 3/6 of the cases did not successfully have malalignment improved when using small bone plates was due to these 3 fractures being chronic in nature. Despite weak evidence, the study’s author still drew conclusions from their research to support the empirical guidelines for whether external coaptation versus surgical stabilisation should be used to manage metabone fractures.

Kapatkin et al. (2000) questioned the accepted empirical guidelines. Although surgery versus conservative management in this study was purely driven by client preference, all cases in the study met the empirical criteria for recommended surgical intervention as highlighted above. The outcomes of these cases, based on clinician and/or owner assessment, were not statistically affected by whether the patients were treated conservatively or surgically. It found that recovery time was longer in the surgically treated group and 4/5 cases treated with IM pins required explantation further down the line. This was a small case series of 25 and there were limitations to accurate follow up of these patients. The majority of assessment in the conservative group was via client questionnaire and the surgical intervention techniques were not standardised.

Kornmayer et al. (2014) looked at the largest pool of cases and contained the most relevant follow up data, looking at radiographic interpretation, clinical outcomes, and outcome measures through computed gait analysis using a treadmill with force plates, however, this was only available for 15/100 cases. A limitation in this paper was that the guidelines set by Manley (1981) were generally obeyed and that only cases with mildly displaced fractures, those that werenon-reconstructible surgically and those that could undergo accurate closed reduction were managed with external coaptation. Dogs with severe displacement, reconstructible articular fractures, and fractures involving the third and fourth metabones were generally managed surgically with dowel pinning, bone plates, external skeletal fixation (ESF), or lag screws. This meant that case treatment selection was not randomised in any way. All cases had a minimum of 4 month follow up with an average of 4 year follow up. Outcomes measured were radiographic (looking for signs of malunion, osteoarthritis (OA), non-union, and synostosis) as well as clinical outcomes of the dog’s lameness graded as either being present or absent. The paper found that clinical outcomes for these patients were generally good in all treatment groups which is contradictory to early papers (Manley, 1981; Muir & Norris, 1997) and lameness often resolved. It showed that radiographic synostosis was more common after surgical intervention but the occurrence of malunion, OA and non-union was not statistically different between the groups. It did show that complications relating to the treatment were more likely to result in malunion and synostosis particularly in the metatarsal bones and that complications are more likely when dealing with open fractures, oblique or comminuted fractures of the metabone base, and severely displaced fractures. It concluded that the empirical guidelines could not be confirmed or refuted and potentially, the fact that the outcomes of the cases in the more displaced/complicated fractures were good, could be interpreted as evidence to support the use of surgical fixation, but this could be considered as confirmation bias.

Bellenger et al. (1981) performed a retrospective study looking specifically at racing greyhounds with racing/training injuries resulting in predominantly single bone fractures of metacarpal 5 and 2). Ten cases of various fracture configuration in other non working pet dogs were initially included but then excluded from the study with no reporting on the treatment or outcomes of these cases. The track injuries studied are potentially unique to racing greyhounds but given there were both surgically and conservatively managed cases (using plaster of Paris), the study was included in this Knowledge Summary. Unfortunately, the main findings of the study were not relevant to the PICO question as no clinical outcomes of lameness were measured and the emphasis was more on return to racing performance which has many other variables than just metabone fracture recovery. In this study 8/14 (57%) of surgically managed cases returned to racing but only 2/8 (20%) conservatively managed cases returned to racing, however, the statistical significance of this is unclear. The radiographic follow up within the study may be of interest as all surgical cases except two that were radiographed too early in their recovery (less than 1 week post surgery) showed good healing whereas the two conservatively managed cases only showed callus formation with no mention of complete healing which may support that conservative management can lead to healing, but alignment and apposition can be compromised resulting in instability and callus formation. There were only two conservatively managed cases with radiographic follow up however and one was a comminuted fracture so callus would be expected in the healing process, therefore this has to be interpreted with this in mind.

Rosselló et al. (2022) published a recent retrospective case study comparing outcomes of open surgical stabilisation (internal fixation) to closed surgical stabilisation (ESF) in metabone fractures of dogs and cats. Although this paper was excluded from this Knowledge Summary due to their being no comparison to conservatively managed cases, it may be of some relevance. The use of ESF and its principles in preserving the soft tissue envelop and blood supply to metabone fracture shares some similarities to the argument for the use of external coaptation in management of these metabone fractures. However, in this study the closed repair group (ESF) showed a significantly greater proportion of delayed healing/non union than the open surgical repair group (12/32 (37.5%) versus 2/31 (6.5%)) and a significantly greater proportion of malalignment (11/32 (34.4%) versus 2/31 (6.5%)). These closed approach findings would correlate with the other papers (Manley, 1981; Muir & Norris, 1997) in this Knowledge Summary that suggest malalignment/malunion is more likely with closed reduction and external coaptation too. Interestingly, these complications were considered minor in this study by Rosselló et al. (2022), since they did not require further interventions, so the assumption is that despite these, the patients regained functionality of the foot, however, clinical outcome/ resolution of lameness is not a reported outcome measure

In conclusion, as can be the case in veterinary science, strong evidence-based treatment guidelines are lacking and there is a need for randomised, prospective, controlled clinical trials in this area. The ethical grounds for this sort of study, however, would be questionable as it would require purposefully selecting not to surgically intervene in cases that meet a consensus for being surgical candidates just to prove or disprove a hypothesis. This could lead to considerable patient morbidity and potential increases in costs for clients. If a study like this was to exist, it may need to be terminated if a pattern of unfavourable outcomes were becoming apparent. Kornmayer et al. (2014) concluded there is not enough evidence to confirm or refute the empirical metabone fracture guidelines so clinical judgement and experience will still be needed until such times that evidence exists for one approach or another. It is worth noting that it is very difficult to provide clear evidence from the literature to support a specific method (conservative or surgical) for metabone fracture treatment as the combinations and variations of the different fractures that can occur in the feet of our patients are vast. With regard to the specific clinical scenario in this Knowledge Summary, non surgical management could be considered in this particular case with outcomes potentially comparable to surgical intervention. The evidence supporting this as a definitive or best treatment (as the owner expressed interest in) is weak, so consideration would have to be given to other variables such as financial feasibility, patient temperament, client compliance as well as the capacity to successfully achieve closed fracture reduction given the perceived instability. Importantly, external coaptation is not a cost free endeavour and repeated dressings and dealing with dressing related complications can become expensive. Complications related to dressings are well documented (Anderson & White, 2000; Meeson et al., 2011) with sometimes catastrophic consequences are possible. Readers should also note that many other studies that were excluded from this Knowledge Summary due to a lack of comparison of surgery versus conservative management of metabone fractures, describe specific surgical repair techniques with reported good outcomes. Modern advances and refinement of osteosynthesis technologies and techniques have also led to better outcomes generally in veterinary treatment of longbone fractures but in particular metabone fractures, especially with the introduction of epoxy putty ESF techniques (Fitzpatrick et al., 2011; De La Puerta et al., 2008), veterinary cuttable plates, smaller locking compression plates’s (Marturello & Perry, 2024) and recent advances in fluoroscopic guided techniques (von Pfeil et al., 2024)and minimally invasive plate osteosynthesis techniques (Piras & Guerrero, 2012).

## Methodology

**Search Strategy:** Caption ..

Databases searched and dates covered:	CAB Abstracts on the OVID interface: 1973 to 2024 Week 33 PubMed on the NCBI interface: 1920 to August 2024
Search strategy:	CAB Abstracts: (dog or dogs or canine*).mp. or exp dogs/ ((metacarp* or metatars* or metabone*) and fracture*).mp. (surgery or ‘surgical repair’ or ‘open reduction’ or ‘closed reduction’ or ‘external fixation’ or ‘internal fixation’ or plat* or pin*).mp. or exp surgery/ (‘external coaptation’ or cast* or bandag* or dressing* or ‘Robert Jones’ or conservative or ‘closed reduction’ or immobilis*).mp. 1 and 2 and 3 and 4 PubMed: dog OR dogs OR canine OR canines (metacarpal OR metatarsal OR metabone OR metabones) AND fracture surgery OR 'surgical repair' OR 'open reduction' OR 'closed reduction' OR 'external fixation' OR 'internal fixation' OR plate OR plating OR pin OR pinning ('external coaptation' OR cast OR bandage OR dressing OR 'robertjones' OR conservative OR 'closed reduction' OR immobilisation) 1 AND 2 AND 3 AND 4
Dates searches performed:	19 Aug 2024

**Exclusion / Inclusion Criteria:** 

Exclusion:	Search results that do not include metabone fractures. Studies not directly comparing conservative and surgical management of metabone fractures. Non English language papers. Studies that summarise or review the literature that was present at that time without any new data or comparisons.
Inclusion:	Papers that contain original data directly comparing outcomes of conservative treatment vs. surgical treatment for cases of metabone fractures.

**Search Outcome:** 

Database	Number of results	Excluded – no metabone fracture involvement	Excluded – did not include both conservative and surgical management of metabone fractures	Excluded – non English language	Excluded – no original data/summary paper	Excluded – non comparative	Total relevant papers
CAB Abstracts	27	12	4	2	4	1	4
PubMed	19	8	6	0	2	0	3
Total relevant papers when duplicates removed							5

## ORCiD

James Phillips: https://orcid.org/0009-0002-7532-4088

## Conflict of Interest

The author declares no conflicts of interest.
